# Exploring evidence of fatigue in survivors of paediatric brain tumours: a systematic review

**DOI:** 10.1192/bjo.2021.799

**Published:** 2021-06-18

**Authors:** Jennifer Wood, Sarah Verity

**Affiliations:** 1 Newcastle University Medical school; 2 Newcastle upon Tyne Hospitals NHS FT, Newcastle University Centre for Cancer

## Abstract

**Aims:**

As the number of survivors of childhood brain tumor grows, fatigue is being increasingly recorded as a long-term consequence of both the cancer itself and the treatment received. Survivors of childhood brain tumour report more significant fatigue than children with other cancers, often impacting all aspects of life, including academic attainment, self-concept and social relationships with peers, leading to reduced health-related quality of life.

This study aimed to systematically evaluate the evidence for fatigue in paediatric brain tumour survivors.

**Method:**

A systematic search using EMBASE, MEDLINE and PsycINFO identified 20 papers meeting the inclusion criteria. Scientific rigor was used throughout by following Scottish Intercollegiate Guidelines Network (2015) guidance for systematic reviews. Quality Assessment of Evidence Rating tool - Fatigue (QAERT) was developed with substantial inter-rater agreement found.

**Result:**

19 of the 20 studies reviewed showed conclusive evidence of fatigue in survivors of paediatric brain tumour. One study offered adequate evidence that there was no difference in levels of fatigue in paediatric cancer survivors, including survivors of paediatric brain tumour, when compared to healthy controls. Three studies found that fatigue was worse in survivors of paediatric brain tumour when compared to survivors of other paediatric cancers

**Conclusion:**

This review provides evidence for the presence of fatigue in survivors of paediatric brain tumour. However, the construct of fatigue was poorly defined throughout, with fatigue associated with physical effects of treatment and fatigue associated with long-term cognitive impairment not distinguished. This poor construct validity, coupled with a lack of comparison groups in 12 of the 20 studies, reduces the generalizability of the data and its usefulness for developing effective psychological interventions. Further research is needed, built on a clear fatigue construct definition, and including well defined exclusion criteria, to provide a sound basis for improving the quality of life of these children.

